# Macular changes following cataract surgery in eyes with early diabetic retinopathy: an OCT and OCT angiography study

**DOI:** 10.3389/fmed.2023.1290599

**Published:** 2023-11-14

**Authors:** Huiping Yao, Zijian Yang, Yu Cheng, Xi Shen

**Affiliations:** Department of Ophthalmology, Ruijin Hospital, Shanghai Jiao Tong University School of Medicine, Shanghai, China

**Keywords:** macular thickness, vascular density, choroidal thickness, optical coherence tomography, diabetic retinopathy, diabetic macular edema, optical coherence tomography angiography

## Abstract

**Background:**

To evaluate changes in macular status and choroidal thickness (CT) following phacoemulsification in patients with mild to moderate nonproliferative diabetic retinopathy (NPDR) using optical coherence tomography.

**Methods:**

In this prospective study, all of the patients underwent uncomplicated phacoemulsification. Retinal superficial capillary plexus vascular density (SCP-VD), macular thickness (MT), and CT were measured pre- and postoperatively.

**Results:**

Twenty-two eyes of 22 cataract patients with mild to moderate NPDR without diabetic macular edema (DME) and 22 controls were enrolled. BCVA increased in two groups at 3 months postoperatively. At 1 and 3 months postoperatively, SCP-VD in the diabetic retinopathy (DR) group significantly increased; changes in SCP-VD in parafovea were significantly greater in the DR group than in the control group. MT and CT in the DR group significantly increased at all visits postoperatively in the fovea and perifovea. Changes in parafoveal MT were significantly greater in the DR group than in the control group at all visits postoperatively. Changes in CT and MT in the fovea were significantly greater in patients with DR than in the controls 1 and 3 months postoperatively.

**Conclusion:**

Uncomplicated phacoemulsification resulted in greater increases in SCP-VD, MT and CT in patients with early DR without preoperative DME than in controls.

## Introduction

Cataract is a leading cause of blindness worldwide (1), and diabetes mellitus (DM) is one of the most common diseases and its prevalence is increasing (2, 3). People with DM are five times more likely to develop cataracts than those without DM (4, 5). Phacoemulsification is the most common, effective, and safe procedures for the treatment of cataract. However, it was reported that cataract surgery was a risk factor for postoperative macular edema and secondary progression in diabetic patients (6). Macular edema and DR progression are the major reasons for poor visual prognosis after cataract surgery in diabetic patients with or without DR (7). While, it has also been suggested that these complications may not be a direct effect of surgery but rather a natural course of disease progression (8–10); therefore, it is clinically meaningful to determine the possible impact of cataract surgery on the occurrence of diabetic macular edema (DME) and/or the progression of DR. This may be related to the timing of cataract surgery as well as the treatment strategy for postoperative ME.

Fluorescent angiography (FA) and indocyanine green angiography (ICGA) are sensitive methods of detecting changes in the retinal and choroidal vasculature. FA is highly sensitive to retinal microvascular fluid leakage (11). However, there are many limitations to the clinical application of FA and ICGA owing to their invasive nature, the risk of allergic reactions, and the inability to achieve a good quantification of measurement. Optical coherence tomography (OCT) is thought to be more sensitive in detecting the presence of ME and is widely used to provide an objective and quantitative assessment of ME (12), providing more detailed images of the retinal and choroidal anatomy. Furthermore, optical coherence tomography angiography (OCTA) can help detect microvascular changes and assess retinal perfusion (13).

The aim of this study was to explore the influence of cataract surgery on macular microvasculature and macular and choroidal thicknesses in patients with mild/moderate DR through follow-up of changes in macular vascular density (VD), macular thickness (MT), and choroidal thickness (CT) using OCT and OCTA to evaluate the influence of phacoemulsification on retinal and choroidal microcirculation and structure.

## Methods

### Study subjects

This prospective observational case–control study included 22 eyes of 22 patients with cataract, who were clinically diagnosed with mild to moderate nonproliferative diabetic retinopathy (NPDR) without DME, and 22 age-matched non-diabetic patients with cataract. All patients underwent complete ophthalmologic examinations, including slit-lamp examination, best-corrected visual acuity (BCVA), dilated fundal examinations, intraocular pressure (IOP), and axial length (AL). Classification and diagnosis of DR and DME were evaluated according to the international clinical disease DR severity scale (14). If a patient underwent surgery on both eyes, the eye that underwent surgery first was included in this study. This study was approved by the Ethical Review Committee of Ruijin Hospital and adhered to the provisions of the Declaration of Helsinki. The consent forms were signed by all patients.

All patients were recruited consecutively from the Ophthalmology Department of Ruijin Hospital between October 2020 and October 2022. The inclusion criteria were: age ≥ 40 years, spherical diopter <−6 D, AL < 26 mm, no history of intraocular surgery or ocular trauma, no history of DME and DR treatment, and no history of glaucoma, uveitis, or other retinal diseases, such as vitreoretinal interface disorders, retinal vein occlusion, retinal artery occlusion, etc.

### Optical coherence tomography measurement

Swept source (SS)-OCT (Triton DRI-OCT, Topcon, Inc., Tokyo, Japan) was used to obtain OCT B-scan and angiography images. Angiography scans (3 × 3 mm) centered on the fovea and 12 radial scans through the center of the fovea were performed to obtain macular OCTA and B scan images. Images with a signal strength index >40 were saved for further analysis.

Superficial capillary plexus vascular density (SCP-VD), MT, and CT values were automatically provided by the built-in software of the SS-OCT device (Topcon FastMap, version 10.13.003.06). The macula was divided into three subfields according to the standard Early Treatment Diabetic Retinopathy Study (ETDRS) grid, as previously reported (15): foveal MT/VD were defined as values in the center circle (1.0 mm); parafoveal MT/CT/VD were defined as the arithmetic average values in the annular subfield with 1.0 mm inside and 3.0 mm outside diameter; and perifoveal MT/CT were defined as the arithmetic average values in the annular subfield with 3.0 mm inside and 6.0 mm outside diameter.

### Surgery

One experienced surgeon performed all phacoemulsification procedures using the Infinity Vision System (Alcon Laboratories, Inc.). No surgery-related complications occurred, and intraocular lens was implanted into the capsular bag during surgery on each patient. The cumulative dissipated energy (CDE) data were collected. Tobramycin and dexamethasone eye drops 4 times per day were administered to all patients for 1 week after surgery, followed by 1.0% prednisolone acetate and 0.5% levofloxacin eye drops 3 times per day for 3 weeks.

### Statistical analysis

Data were analyzed using GraphPad Prism 7.0 (GraphPad Software, CA) and expressed in the form of mean ± standard deviation (SD). The normal distribution of continuous variables was tested using D’Agostino & Pearson normality test. The differences between two groups were compared using independent t test. MT, CT, and SCP-VD at the four visits were compared using the repeated measures analysis of variance (ANOVA) with Bonferroni correction. Categorical variables were analyzed using Fisher’s exact test. In addition, the repeated measures analysis of variance for the four visits with the inclusion of the CDE covariant (ANCOVA) was performed with SPSS software version 21.0 (SPSS, Inc., IL, Chicago, United States) to assess the effect of CDE on variations in OCTA parameter. *p* < 0.05 was considered statistically significant.

## Results

### Demographic and clinical characteristics

A total of 44 eyes of 44 participants were enrolled in the study; the DR group included 22 patients with mild to moderate NPDR (13 males and 9 females) and the control group included 22 nondiabetic patients (12 males and 10 females). There were no significant differences in age, sex, BCVA, IOP, CDE or AL between the two groups at baseline. The DR group included 3 patients with mild NPDR and 19 with moderate NPDR. BCVA at 3 months postoperatively increased in DR and control groups (all *p* < 0.001). There was no significant difference in BCVA between the two groups at 3 months postoperatively (*p* = 0.615). In the control group, the IOP at 1 week, 1 month, and 3 months postoperatively was lower than at baseline (*p* < 0.001, p < 0.001, and *p* = 0.003, respectively), and in the DR group, the IOP at 1 week, 1 month, and 3 months postoperatively was also lower than at baseline (*p* < 0.001, *p* < 0.001, and *p* = 0.006, respectively) (Table 1). One patient progressed from mild NPDR to moderate NPDR, while the other patients showed no progression in the stage of DR and no occurrence of DME at the last visit.

**Table 1:** Demographic and clinical characteristics.

		Controls (*n* = 22)	NPDR (*n* = 22)	*p*-value
Age, years (mean ± SD)		65.9 ± 1.43	65.1 ± 1.68	0.727
Gender (n)	Female	9	10	>0.999
	Male	13	12	
BCVA, log MAR (mean ± SD)	Baseline	0.75 ± 0.19	0.77 ± 0.21	0.710
	3 Mo postop	0.07 ± 0.13	0.09 ± 0.1	0.615
IOP, mmHg (mean ± SD)	Baseline	16.3 ± 2.31	16.1 ± 2.7	0.776
	1 Wk Postop	12.4 ± 3.46	12.9 ± 2.6	0.59
	1 Mo Postop	13.8 ± 1.92	13.4 ± 2.05	0.522
	3 Mo Postop	14.2 ± 2.05	14.1 ± 1.86	0.945
Axial length, mm(mean ± SD)		23.25 ± 0.94	23.31 ± 0.85	0.824
CDE(mean ± SD)		6.61 ± 4.9	5.86 ± 3.1	0.546

NPDR, nonproliferative diabetic retinopathy; BCVA, best-corrected visual acuity; IOP, intraocular pressure; Wk, week; Postop, postoperatively; Mo, month; CDE, cumulative dissipated energy; SD, standard deviation.

### Superficial capillary plexus vascular density

The microvasculature parameters obtained using OCTA at all visits are presented in Table 2. At baseline, there was no significant difference in foveal SCP-VD between the DR group and the control group (*p* = 0.446); however, parafoveal SCP-VD in the DR group was lower than that in the control group (*p* = 0.005).

**Table 2:** Superficial capillary plexus vascular density of the two groups at four visits (mean ± SD).

		Baseline	Postop						*p* value
			1 Wk Postop		1 Mo Postop		3 Mo Postop		
		VD	VD	*p* value	VD	*p* value	VD	*p* value	
DR group (%)	Fovea	18.65 ± 2.05	18.23 ± 1.72	0.54	18.77 ± 1.64	>0.999	18.48 ± 1.78	>0.999	0.216
	Parafovea	46.63 ± 1.74	47.18 ± 1.35	0.339	49.11 ± 1.69	<0.001	48.67 ± 1.38	<0.001	<0.001
Control group (%)	Fovea	19.2 ± 2.6	18.6 ± 2.9	0.478	19.38 ± 3.01	>0.999	19.29 ± 2.9	>0.999	0.213
	Parafovea	48.28 ± 2	48.58 ± 1.86	>0.999	49.44 ± 1.69	0.122	49.05 ± 1.45	0.369	0.027

Wk, week; Postop, postoperatively; Mo, month; DR, diabetic retinopathy; VD, vascular density.

In the DR group, there was no significant difference between postoperative foveal SCP-VD and baseline foveal SCP-VD at 1 week, 1 month, 3 months postoperatively; parafoveal SCP-VD at 1 week after surgery was not significantly different from baseline; parafoveal SCP-VD increased significantly at 1 and 3 months after surgery compared with baseline (Table 2). In the control group, SCP-VD after surgery was not significantly different from baseline at all visits postoperatively in fovea and parafovea (Table 2).

In DR group, there existed significant difference in parafoveal SCP-VD between 1 week and 1 months postoperatively (*p* < 0.001), and the same was true between 1 week and 3 months postoperatively (*p* < 0.001); other than that, there were no significant differences in SCP-VD between any two time points postoperatively in both fovea and parafovea (all *p* > 0.05). In the control group, there were no significant differences in SCP-VD between any two time points postoperatively in both fovea and parafovea (all *p* > 0.05).

Changes in SCP-VD in the DR group were significantly greater than those in the control groups 1 and 3 months postoperatively in the parafovea. Changes in SCP-VD in the DR group were not significantly different from those of the control group in the fovea at 1 week, 1 month and 3 months postoperatively (Table 3).

**Table 3:** Changes in superficial capillary plexus vascular density after cataract surgery (mean ± SD).

		1 Wk Postop			1 Mo Postop			3 Mo Postop		
		Control	DR	*p* value	Control	DR	*p* value	Control	DR	*p* value
VD (%)	Fovea	−0.59 ± 1.51	−0.42 ± 1.1	0.671	0.18 ± 1.79	0.11 ± 1.67	0.895	0.09 ± 2.3	−0.17 ± 1.68	0.673
	Parafovea	0.3 ± 1.45	0.56 ± 1.3	0.529	1.16 ± 2.17	2.49 ± 1.96	0.038	0.77 ± 1.82	2.05 ± 1.35	0.011

Wk, week; Postop, postoperatively; Mo, month; DR, diabetic retinopathy; VD, vascular density.

ANCOVA test did not show relationships between CDE and variations in SCP-VD in both DR and control groups. After adjusting for CDE, parafoveal SCP-VD increased significantly at 1 and 3 months after surgery compared with baseline in the DR group (Supplementary Tables S1, S2).

### Macular thickness

At baseline, there was no significant difference between the DR and control group in the foveal, parafoveal, and perifoveal MT (*p* = 0.637, 0.927, and 0.773, respectively).

The MT of the DR group significantly increased at 1 week, 1 month, and 3 months postoperatively compared with baseline in all subfields; in the control group, the MT significantly increased at 1 week, 1 month, and 3 months postoperatively in fovea and parafovea, and significantly increased at 1 and 3 months postoperatively compared with baseline in perifovea (Table 4).

**Table 4:** The macular thickness of the two groups at four visits (mean ± SD).

		Baseline	Postop						*p* value
			1 Wk Postop		1 Mo Postop		3 Mo Postop		
		MT	MT	*p* value	MT	*p* value	MT	*p* value	
Control (μm)	Fovea	219.3 ± 19.98	228.8 ± 21.38	0.004	244 ± 25.42	<0.001	236.5 ± 21.53	<0.001	<0.001
	Parafovea	292.2 ± 20.28	299.3 ± 18.54	0.014	311 ± 15.14	<0.001	303.9 ± 18.51	0.002	<0.001
	Perifovea	264.4 ± 16.03	271.2 ± 16.83	0.067	281.5 ± 23.78	<0.001	271.2 ± 17.45	<0.001	<0.001
DR (μm)	Fovea	222.3 ± 21.85	235.2 ± 20.21	<0.001	262.1 ± 31.15	<0.001	257.1 ± 28.92	<0.001	<0.001
	Parafovea	291.5 ± 25.07	306.9 ± 18.8	<0.001	318.8 ± 21.12	<0.001	314.1 ± 19.2	0.001	<0.001
	Perifovea	266 ± 18.97	277.9 ± 18.11	0.022	290 ± 20.04	<0.001	286.3 ± 17.87	<0.001	<0.001

Wk, week; Postop, postoperatively; Mo, month; DR, diabetic retinopathy; MT, macular thickness.

In DR group, there existed significant difference between MT at 1 week and 1 month postoperatively in the fovea, parafovea, and perifovea (*p* < 0.001, *p* < 0.001, and *p* < 0.001, respectively); the same was true between MT at 1 and 3 months postoperatively in the fovea, parafovea (*p* = 0.02 and 0.02, respectively) and between MT at 1 week and 3 months postoperatively in the fovea, parafovea, and perifovea (*p* = 0.001, *p* = 0.008, and *p* < 0.001, respectively). In control group, there existed significant difference between MT at 1 week and 1 month postoperatively in the fovea, parafovea, and perifovea (*p* < 0.001, *p* < 0.001, and *p* = 0.007, respectively); the same was true between MT at 1 and 3 months postoperatively in the parafovea, perifovea (*p* = 0.021 and 0.032, respectively).

Changes in MT in the DR group were significantly greater than those in the control group at 1 and 3 months postoperatively in the fovea and parafovea, and the same was true for MT in the parafovea at 1 week postoperatively and in the perifovea at 3 months postoperatively (Table 5).

**Table 5:** Changes of macular thickness after cataract surgery (mean ± SD).

		1 Wk Postop				1 Mo Postop		3 Mo Postop		
		Control	DR	*p* value	Control	DR	*p* value	Control	DR	*p* value
MT (μm)	Fovea	9.5 ± 11.5	13.0 ± 11.1	0.323	24.68 ± 20.01	39.86 ± 19.34	0.014	17.23 ± 13.15,	34.86 ± 17.68	0.001
	Parafovea	7.16 ± 9.69	15.38 ± 13.91	0.028	18.87 ± 12.35	27.24 ± 12.64	0.032	11.69 ± 12.87	22.55 ± 13.95	0.01
	Perifovea	6.75 ± 11.38	11.9 ± 17.02	0.245	17.11 ± 17.32	24.06 ± 17.32	0.177	6.75 ± 6.21	20.38 ± 16.81	0.001

Wk, week; Postop, postoperatively; Mo, month; DR, diabetic retinopathy; MT, macular thickness.

**Figure 1:**
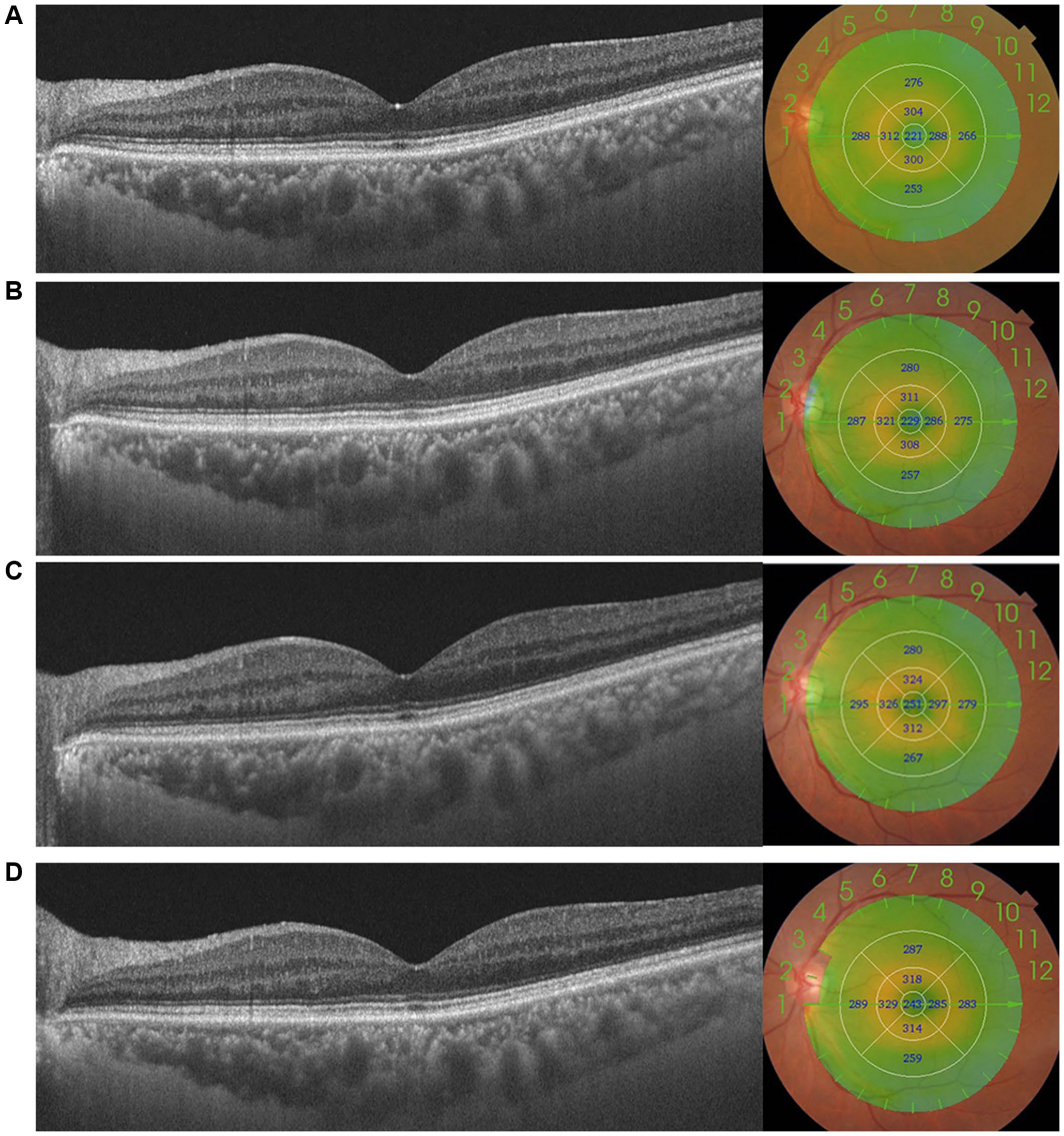
This figure shows the changes in macular thickness (MT) preoperatively **(A)** and 1 week **(B)**, 1 month **(C)**, and 3 months **(D)** postoperatively in a patient with moderate DR. MT in the central 1,000 μm increased after surgery, increasing to 251 μm, and absent of pseudophakic cystoid macular oedema (PCME).

The distribution of foveal MT of the control and DR groups is shown in Table 6. The proportion of foveal MT >250 μm reached its maximum, up to 68.2%, at 1 month postoperatively and was 54.5% at 3 months postoperatively in the DR group, while in the control group it was highest at 1 month (40.9%). There was no significant difference in the distribution of foveal MT between the two groups (Table 6). No patient was found to develop to pseudophakic cystoid macular edema (PCME) during the follow-up period (Figure 1).

**Table 6:** Distribution of foveal macular thickness of the two groups at four visits (mean ± SD).

Foveal MT (μm)	Baseline		1 Wk Postop		1 Mo Postop		3 Mo Postop	
	≤250	>250	≤250	>250	≤250	>250	≤250	>250
DR(*n*, %)	20 (90.9%)	2 (9.1%)	16 (72.7%)	6 (27.3%)	7 (31.8%)	15 (68.2%)	10 (45.5%)	12 (54.5%)
Control(*n*, %)	22 (100%)	0	19 (86.4%)	3 (13.6%)	13 (59.1%)	9 (40.9%)	16 (72.7%)	6 (27.3%)
*p*-value	0.488		0.457		0.13		0.124	

Wk, week; Postop, postoperatively; Mo, month; MT, macular thickness.

### Choroidal thickness

At baseline, there were no significant difference between the DR and control groups in foveal, parafoveal and perifoveal CT (*p* = 0.376, 0.203, and 0.107, respectively).

The CT of the DR group increased significantly at 1 week, 1 month and 3 months postoperatively in the fovea and perifovea and 1 month and 3 months postoperatively in the parafovea compared with baseline (Table 7). In the DR group, there existed significant difference in CT between 1 week and 1 month postoperatively in the fovea (*p* = 0.019); other than that, there were no significant differences in CT between any two time points postoperatively in both fovea and parafovea (all *p* > 0.05). In the control group, there were no significant differences in CT between any two time points in all subfields (all *p* > 0.05).

**Table 7:** The choroidal thickness of the two groups at four visits (mean ± SD).

		Baseline	Postop						*p*-value
			1 Wk Postop		1 Mo Postop		3 Mo Postop		
		CT	CT	*p*-value	CT	*p*-value	CT	*p*-value	
Control (μm)	Fovea	222.6 ± 104.5	226.8 ± 107.8	0.57	228.8 ± 110.1	>0.999	229.5 ± 112.4	0.273	0.346
	Parafovea	220 ± 101.2	224.4 ± 105.9	0.667	228.6 ± 106.9	0.115	226.5 ± 105.4	0.21	0.022
	Perifovea	193.1 ± 84.98	198.3 ± 87.8	0.694	202.7 ± 87.35	0.066	201.8 ± 89.17	0.131	0.015
DR (μm)	Fovea	199.6 ± 59.2	211.4 ± 66.57	0.041	219.5 ± 64.53	<0.001	216.3 ± 62.61	<0.001	<0.001
	Parafovea	188.1 ± 56	199.2 ± 62.87	0.064	204.3 ± 57.48	<0.001	203.4 ± 57.48	0.001	<0.001
	Perifovea	158.8 ± 48.44	168.7 ± 53.78	0.039	174.7 ± 49.07	<0.001	172.4 ± 48.2	0.001	<0.001

Wk, week; Postop, postoperatively; Mo, month.

Changes of CT in the DR group were significantly greater than those of the control group at 1 and 3 months postoperatively in the fovea, and at 3 months postoperatively in the parafovea (Table 8).

**Table 8:** Changes in choroidal thickness after cataract surgery (mean ± SD).

		1 Wk Postop			1 Mo Postop			3 Mo Postop		
		Control	DR	*p* value	Control	DR	*p* value	Control	DR	*p* value
CT (μm)	Fovea	4.25 ± 11.4	11.77 ± 18.42	0.111	6.21 ± 28.81	19.83 ± 12.06	0.035	6.93 ± 15.29	16.63 ± 13.38	0.031
	Parafovea	4.43 ± 12.5	11.13 ± 18.62	0.169	8.64 ± 15.95	16.24 ± 16.37	0.126	6.51 ± 13.54	15.34 ± 14.71	0.045
	Perifovea	5.17 ± 14.78	9.89 ± 15.32	0.305	9.56 ± 16.1	15.94 ± 10	0.121	8.67 ± 16.42	13.6 ± 14.23	0.293

Wk, week; Postop, postoperatively; Mo, month; DR, diabetic retinopathy; CT, choroidal thickness.

## Discussion

Breakdown of the blood-retina barrier (BRB) can lead to the development of DR and ME (16). On the other hand, choroid is the only source of metabolic exchange for the avascular fovea (17). Postoperative inflammation may aggravate choroidal microvascular lesions, in addition to the retinal microcirculation (18). It is of great significance to clarify retinal and choroidal changes and the relationship with the occurrence of ME and deterioration of DR after cataract surgery in patients with DR, especially in early DR without DME, in which the occurrence of ME and the progression of retinopathy are crucial to postoperative visual prognosis.

Here, we report a significant increase in MT within 3 months after cataract surgery in a specific population with mild to moderate DR. George et al. observed hyperfluorescence of the macula and optic disc postoperatively on fluorescein angiography in the surgical eyes, which was greater than that in the nonsurgical eyes (19). Moreover, increased macular thickness has previously been reported in surgical diabetic eyes (18, 20), which is similar to the results of our research, although the population studied was not entirely consistent. We also observed a significant increase in CT after surgery in the DR group. This finding is consistent with the results of previous studies (15, 21–23). In addition, MT in the parafovea, and perifovea increased significantly in the control group at 1 week, 1 month, and 3 months postoperatively; while CT in the parafovea and perifovea slightly increased, though which was not significantly thicker than baseline in the control group at 1 month postoperatively. This indicates that surgical trauma itself induces a rapid increase in macular and choroidal thicknesses. To the best of our knowledge, this is the first observational follow-up study about macular microvasculature changes after phacoemulsification in this specific population of patients with mild to moderate DR using the OCTA-based VD calculation and analysis. SCP-VD in the parafoveal macula increased significantly at 1 and 3 months postoperatively compared with baseline in patients with DR in this study. This is consistent with a previous study that reported that vascular densities increased in both the SCP and deep capillary plexus (DCP) after cataract surgery (24–26). This may be due to postoperative inflammation. Inflammation is suspected to be involved in cataract surgery-mediated retinal complications. Cataract surgery induces markedly upregulated expression of IL-1 and CCL2 genes in the retina and choroid (27). In animal studies, breakdown of the inner BRB was observed after lens aspiration (28). And it was reported that decreased IOP resulted in an increase in leukocyte flow speed and choroidal thickness in the macula (22, 29). IOL decreased significantly after surgery in our study, which may be another cause of the increased VD and CT postoperatively.

What is more noteworthy is that, in our study, changes in foveal/parafoveal MT and foveal CT in patients with DR were significantly greater than those in controls at 1 and 3 months postoperatively; changes in SCP-VD in patients with DR were significantly greater than those in the control group in the parafovea at 1 and 3 months postoperatively. Presumably, it was suggested that cataract surgery might increase the risk of macular edema in patients with mild/moderate DR compared with patients without diabetes, which may be due to defects in blood-retinal barrier function in those patients with more advanced vascular changes caused by microvascular destruction in DR than in normal individuals. This finding is consistent with those of other studies. It has been reported that the retinal thickness of diabetic patients has a significantly increasing trend compared with that in non-diabetic patients (30). The presence of diabetes was a risk factor of pseudophakic macular edema (PME) and the risk increased approximately linearly with the severity of retinopathy (31). However, previous studies found no significant difference in the magnitude of changes after cataract surgery between diabetic and non-diabetic patients (32). This is inconsistent with our results and may be due to the inclusion of different patient population, and the patients enrolled in their study had less severe DR.

As is well-known, inflammation plays a role in retinal microvasculature damage and exists at different stages of DR. It plays a crucial role in the pathogenesis of DR by increasing retinal vascular permeability and promoting neovascularization, which leads to the occurrence and development of DME and DR (33–35). Diabetic patients exhibit an overall higher level of inflammatory activity than non-diabetic patients, according to previous clinical and laboratory studies (36–38). The expression of vascular endothelial growth factor (VEGF), which may be involved in increased vascular permeability and angiogenesis, is upregulated in the diabetic retina and choroid (39). And the levels of VEGF and IL-6 in aqueous humor are significantly correlated with macular edema severity in diabetic patients (40). The significantly greater changes in MT, foveal CT, and parafoveal VD observed at 1 and 3 months postoperatively in the DR group than in the control group may be due to the inflammation present in DR. Preexisting inflammatory activity caused by DR, as well as the surgery-induced inflammatory process, might be the cause of increased VD, MT, and CT in patients with mild/moderate NPDR in the present study.

In addition to postoperative inflammation, it was reported that anterior–posterior (AP) traction induced by the surgery procedure might also be a risk factor for macular edema after cataract surgery, and AP traction could disrupt the macular microcirculation, including blood–retinal barrier disfunction and increasing vascular permeability (41). In this study, MT and SCP-VD peaked 1 month postoperatively. Compared to macular thickening caused solely by mechanical traction itself during the surgery, the disrupted macular microcirculation induced by surgery is more likely to be the direct cause of macular thickening. Furthermore, several studies have investigated the relationship between CDE and changes in retinal microcirculation after surgery. In some studies, patients with higher CDE values were reported to have a more significant reduction in VD after surgery (42, 43). In another study, it was found that changes in VD after surgery were not significantly correlated with CDE; therefore, they concluded that CDE had little effect on retinal microcirculation (32). This is consistent with our results. In the present study, there was no significant difference between the two groups, and ANCOVA test did not show relationships between CDE and variations in SCP-VD, which may imply that CDE had little effect on postoperative VD changes. There is some controversy among studies, and this may be due to the inclusion of different patient population and difference in levels of CDE between studies. Presumably, the CDE recorded in the present study was relatively small and insufficient to have a significant effect on OCT parameters. Randomized controlled trials with a large sample size should be needed in future.

In our study, MT, SCP-VD, and CT peaked 1 month after surgery. Previous reports have also shown that the incidence of ME peaks approximately 4–6 weeks after uneventful cataract surgery (44, 45). Temporally, the increases in CT VD and MT seemed to coincide with the timing of the previously reported appearance of ME after surgery. Presumably, postoperative inflammation had the greatest impact on MT, SCP-VD, and CT at 1 month after surgery. From the point of view of time, it seems that choroidal and retinal microcirculation disorders might both be likely to be associated with the increase in MT postoperatively. Nevertheless, further research is required to determine whether the choroid is involved in the development of PME.

In this study, none of the patients developed to DME or PCME during the follow-up period. Foveal MT increased significantly after cataract surgery; foveal MT of more than 250 μm was as high as 68.2% at 1 month postoperatively. Central subfield thickness (CST), which is defined as average retinal thickness in the central 1,000 μm, corresponds to the foveal MT in this study, and 250 μm is usually the CST threshold value for diagnosing DME (46); however, no patients developed DME during the period of follow-up in our study. And in this study, BCVA of patients with DR was equivalent to that of nondiabetic individuals after surgery; therefore, increasing foveal MT could be considered a subclinical macular thickness thickening that did not affect visual acuity and was not a support of PCME, which is consistent with a previous study (10). In addition, DME and PCME after surgery were not be observed in this study, which might be due to the small size and short follow-up period. Moreover, it was reported that the risk of PME in patients with DR is high and increases with the severity of DR (31); preoperative noncenter-involved DME or a history of DME treatment may increase the risk of center-involved DME at 16 weeks after surgery (47). This study enrolled the patients with early DR and without a history of DME, which may be another reason for these observations.

Furthermore, at baseline, the parafoveal VD of DR patients was lower than that of the controls. It was previously reported that VD decreased in patients with DR without macular edema, and that VD parameters decreased with increasing DR severity (48). The foveal VD of patients with DR in our study was slightly lower than that of the controls, although the difference was not significant. This may be due to the small sample size or the fact that the population enrolled in our study included cataract patients with mild/moderate NPDR without preoperative DME, which does not reflect the full spectrum of patients with DR. However, there was no significant difference in MT between the DR and control groups at baseline. This may indicate that microvasculature changes occur before macular structural changes in patients with early DR.

This study had a few limitations. The sample size was relatively small, but the participants were homogeneous in terms of degree of retinopathy, ethnicity and sex, reducing some confounding effects. Moreover, the follow-up period was too short that we cannot observe the long-term effects on retina vessels and structural changes. Long-term follow-up is required to determine the duration of the increase in MT, CT, and VD after cataract surgery and its long-term relationship with the progression of DR severity and incidence of DME, which may be suggestive of a higher incidence of ME in this population. Future studies should enroll a larger sample of participants with longer follow-up periods.

## Conclusion

Uncomplicated phacoemulsification results in a greater increase in MT, SCP-VD, and CT in patients with mild/moderate NPDR without preoperative DME than in controls. This may be suggestive of a predisposition to the occurrence of macular edema in this population, which may be due to the preexisting inflammatory activity caused by DR as well as cataract surgery trauma. However, the short-term postoperative visual prognosis of cataract patients with mild to moderate NPDR without preoperative DME is the same as that of healthy patients in this study.

## Data availability statement

The original contributions presented in the study are included in the article/Supplementary material, further inquiries can be directed to the corresponding author.

## Ethics statement

The studies involving humans were approved by the Ethical Review Committee of Ruijin Hospital. The studies were conducted in accordance with the local legislation and institutional requirements. The participants provided their written informed consent to participate in this study.

## Author contributions

HY: Conceptualization, Data curation, Formal analysis, Investigation, Methodology, Writing – original draft. ZY: Data curation, Investigation, Writing – review & editing. YC: Methodology, Supervision, Validation, Writing – review & editing. XS: Conceptualization, Data curation, Investigation, Methodology, Supervision, Validation, Writing – review & editing.
