# Supplementary material for: Macular changes following cataract surgery in eyes with early diabetic retinopathy: an OCT and OCT angiography study
Source: Front Med (Lausanne). 2023 Nov 14;10:1290599. doi: 10.3389/fmed.2023.1290599 (PMC10682095; doi:10.3389/fmed.2023.1290599)
Supplement: Supplementary file 1 [file Table_1.DOCX]

Supplementary Table 1 Comparisons of superficial capillary plexus vascular density at four visits after adjusting for CDE in the DR group

|  | **1 Wk Postop** VS **baseline** | **1 Mo Postop VS baseline** | | **3 Mo Postop** VS **baseline** | **1 Wk** VS  **1 Mo Postop** | **1 Wk** VS  **3 Mo Postop** | **1 Mo** VS  **3 Mo Postop** | **F/P value** | **CDE** |
| --- | --- | --- | --- | --- | --- | --- | --- | --- | --- |
|  | **P value** | **P value** | **P value** | | **P value** | **P value** | **P value** |  | **F/P value** |
| fovea | 0.576 | 1.000 | 1.000 | | 0.116 | 1.000 | 0.128 | 0.467/0.567 | 0.112/0.827 |
| parafovea | 0.347 | 0.000 | 0.000 | | 0.000 | 0.000 | 1.000 | 13.25/0.000 | 1.898/0.156 |

CDE, cumulative dissipated energy; Wk, week; Postop, postoperatively; Mo, month; DR, diabetic retinopathy; VD, vascular density

Supplementary Table 2 Comparisons of superficial capillary plexus vascular density at four visits after adjusting for CDE in the control group

|  | **1 Wk Postop** VS **baseline** | **1 Mo Postop** VS **baseline** | **3 Mo Postop** VS **baseline** | **1 Wk** VS  **1 Mo Postop** | **1 Wk** VS  **3 Mo Postop** | **1 Mo** VS  **3 Mo Postop** | **F/P value** | **CDE** |
| --- | --- | --- | --- | --- | --- | --- | --- | --- |
|  | **P value** | **P value** | **P value** | **P value** | **P value** | **P value** |  | **F/P value** |
| fovea | 0.451 | 1.000 | 1.000 | 0.157 | 0.687 | 1.000 | 1.684/0.484 | 1.368/0.261 |
| parafovea | 1.000 | 0.137 | 0.402 | 0.127 | 1.000 | 1.000 | 2.106/0.364 | 0.411/0.714 |

CDE, cumulative dissipated energy; Wk, week; Postop, postoperatively; Mo, month; DR, diabetic retinopathy; VD, vascular density
